# Overexpression of cytosolic NADP‐malic enzyme 1 from the common ice plant enhances water‐deficit and high‐light stress tolerance by modulating water‐use efficiency and flavonoid biosynthesis

**DOI:** 10.1111/tpj.70968

**Published:** 2026-06-06

**Authors:** Sang Hun Kim, Dong Yeol Song, Chan Hui Lee, Seo Young Yang, John C. Cushman, Sung Don Lim

**Affiliations:** ^1^ Molecular Plant Physiology Laboratory, Department of Applied Plant Sciences, Graduate School Sangji University Wonju 26339 Republic of Korea; ^2^ Department of Plant & Environmental New Resources, College of Life Sciences Kyung Hee University Yongin 17104 Republic of Korea; ^3^ Department of Biology Education, Teachers College and Institute for Phylogenomics and Evolution Kyungpook National University Daegu 41566 Republic of Korea; ^4^ Department of Biochemistry, Molecular Biology & Biotechnology University of Nevada – Reno Reno Nevada 89557‐0330 USA

**Keywords:** Crassulacean acid metabolism, *Mesembryanthemum crystallinum*, NADP‐malic enzyme, water‐deficit stress tolerance, *Arabidopsis thaliana*, flavonoids

## Abstract

Innovative strategies are essential to enhance crop resilience against drought and heat stress intensified by climate change. Crassulacean acid metabolism (CAM) is a specialized photosynthetic pathway that improves water‐use efficiency (WUE) by shifting CO_2_ fixation to the nighttime. The common ice plant (*Mesembryanthemum crystallinum*) utilizes NADP‐malic enzymes (ME) for malate decarboxylation during its facultative CAM transition. In this study, we characterized the cytosolic *McNADP‐ME1* enzyme, which is highly expressed in the ice plant under water‐deficit stress. Transgenic *Arabidopsis thaliana* plants overexpressing *McNADP‐ME1* exhibited reduced stomatal density, size, and conductance, leading to enhanced instantaneous WUE. Although these modifications resulted in reduced biomass and seed yield under low‐light conditions, the transgenic lines showed significantly improved survival and growth under both acute and chronic water‐deficit stress. Additionally, *McNADP‐ME1* overexpression conferred improved vegetative growth under high‐light stress conditions. Notably, *McNADP‐ME1* overexpression upregulated many genes within the flavonoid biosynthetic pathway, resulting in a marked increase in total flavonoid content. These flavonoids acted as effective antioxidants that facilitated the scavenging of reactive oxygen species (ROS), thereby reducing oxidative damage and malondialdehyde (MDA) levels under both water‐deficit and high‐light stress conditions. These results demonstrate that cytosolic *McNADP‐ME1* is a key enzyme for conferring water‐deficit stress tolerance by integrating stomatal‐mediated water conservation with flavonoid‐driven ROS‐scavenging mechanisms.

## Significance Statement

To sustain agricultural productivity in the face of future climate changes, innovative strategies are required to enhance crop resilience and tolerance to increasingly hot and dry conditions. Here, we report the beneficial effects of overexpression of *McNADP‐ME1* that significantly improved vegetative biomass and seed production in *Arabidopsis thaliana* under water‐deficit stress conditions through decreased transpiration and increased instantaneous water‐use efficiency as well as through increased ROS‐scavenging flavonoid accumulation, thereby improving water‐deficit stress tolerance.

## INTRODUCTION

Increasing agricultural production is a critical issue to ensure a sufficient food supply for the growing global population. According to the United Nations, the global population is projected to reach 8.5 billion by 2030 and 9.7 billion by 2050 (United Nations, 2019), underscoring the necessity for enhancing agricultural output. However, modeling of global temperature increases predicts increased drought, heat, and combined heat and drought events in the future (IPCC, 2023; Mittler et al., 2025; Tripathy et al., 2023; Yin et al., 2022). These climatic changes reduce soil moisture and increase evaporative demands that in turn diminish major crop yields across all regions globally (Arora, 2019; Irmak et al., 2022; Lesk et al., 2022; Ray et al., 2019). Over the past 40 years, drought is estimated to have caused a loss of 1820 million Mg in major crops such as maize and wheat globally, with an additional 1190 million Mg lost due to heat waves (Lesk et al., 2016). Plants respond to drought stress through diverse molecular, cellular, and physiological mechanisms. However, the overproduction of ROS during such stresses causes cellular damage and death, which ultimately reduces crop yield (Verma et al., 2019). To overcome these challenges, strategies focused on maximizing water‐use efficiency and stress resilience are deemed crucial.

Plants have evolved multiple physiological and metabolic mechanisms to mitigate environmental stress such as drought or heat. During periods of such stress, ROS are generated as by‐products of oxygen reduction, including superoxide radicals, hydrogen peroxide (H_2_O_2_), hydroxyl radicals (OH•), and singlet oxygen (^1^O_2_), which are harmful to plants as they damage cellular organelles (Mittler et al., 2004; Sharma et al., 2012). These oxygen radicals impact proteins and lipids, leading to cellular damage and death (Ayala et al., 2014; Suzuki, 2015). Therefore, enhancing the antioxidant activity of plants is essential to reduce damage caused by ROS. H_2_O_2_ and O_2_^−^ and related components also function as secondary messengers that regulate hormone signaling, growth, development, cell death, and stress responses (Choi et al., 2016; Gilroy et al., 2014; Mittler et al., 2022).

Crassulacean acid metabolism (CAM) is a photosynthetic adaptation that significantly reduces water loss, enabling plants to thrive in environments with limited water availability by optimizing water‐use efficiency during carbon fixation (Borland et al., 2011; Winter et al., 2015; Yang et al., 2015). CAM plants typically conduct primary net CO_2_ fixation at night via phospho*enol*pyruvate (PEP) carboxylase (PEPC) to form oxalacetate, which is then converted to malate by NAD(P)‐malate dehydrogenase [NAD(P)‐MDH] and stored in large vacuoles as malic acid. During the day, the stored malic acid is decarboxylated and refixed by ribulose‐1,5‐bisphosphate carboxylase/oxygenase (RUBISCO), which results in the formation of sugars and starch via the Calvin–Benson–Bassham (CBB) cycle of C_3_ photosynthesis, gluconeogenesis, and starch biosynthesis. Depending upon the CAM species, prevailing environmental conditions, and malate storage capacity, the stomata can remain closed for all or part of the day, which improves WUE (Borland et al., 2011; Cushman & Bohnert, 1999).

Malate decarboxylation in CAM plants is catalyzed by multiple malic enzyme isoforms with distinct subcellular localizations and cofactor specificities. Decarboxylation of malate can occur via mitochondrial NAD‐dependent malic enzyme (NAD‐ME), which produces pyruvate that is regenerated by pyruvate orthophosphate dikinase (PPDK) to form phospho*enol*pyruvate (PEP), which then enters the gluconeogenesis pathway. In CAM species such as *Kalanchoë fedtschenkoi*, mitochondrial NAD‐ME plays an important role in malate decarboxylation and circadian regulation of CAM (Dever et al., 2015). RNAi‐mediated knock‐down of the mitochondrial *KfNAD‐ME1* gene or PPDK in *K. fedtschenkoi* resulted in a loss of CAM CO_2_ fixation and reduced circadian clock‐controlled phosphorylation of PEPC in the dark, thereby disrupting the circadian cycles of PEPC phosphorylation. The oscillation in the transcript abundance patterns of core circadian clock genes also became arrhythmic in the *KfNAD‐ME1* knock‐down line (Dever et al., 2015). NAD‐ME decarboxylation of malate within the mitochondria generates pyruvate, which sustains carbon metabolism through the tricarboxylic acid (TCA) cycle and when exported to the cytosol, it fuels gluconeogenesis and facilitates phosphate/proton (Pi/H^+^) homeostasis (Daems et al., 2024).

Alternatively, malate decarboxylation can be catalyzed by cytosolic NADP‐dependent malic enzyme (NADP‐ME), which is particularly relevant in certain CAM species and during the daytime decarboxylation phase. In the common ice plant (*Mesembryanthemum crystallinum*), a facultative CAM model species, NADP‐ME activity is localized to the cytosol and increases substantially during the C_3_‐to‐CAM transition induced by salinity or water‐deficit stress (Cushman et al., 2008; Winter et al., 1982). Cytosolic NADP‐ME catalyzes the oxidative decarboxylation of malate to produce pyruvate, CO_2_, and NADPH in the cytosol during the daytime. This reaction provides three critical metabolic advantages: (1) localized CO_2_ supply for RUBISCO carboxylation with reduced stomatal opening, (2) NADPH generation for biosynthetic pathways and redox homeostasis, and (3) pyruvate/PEP provision for gluconeogenesis and plastidic metabolism (Cushman et al., 2008; Hausler et al., 2000; Winter et al., 1982). The stress‐inducible nature of cytosolic NADP‐ME in *M. crystallinum*, combined with its capacity to generate both carbon intermediates and reducing equivalents, makes it a particularly attractive candidate for engineering stress tolerance in C_3_ crops. A third decarboxylation pathway involves the combined action of NAD(P)‐MDH, which converts malate to OAA, and PEP carboxykinase (PEPCK), which converts OAA to PEP (Lim et al., 2025). Lastly, co‐adaptive traits associated with CAM aid in reducing water loss and increasing water storage capacity, enhancing the plant's ability to survive in environments with reduced or seasonally limited precipitation patterns (Niechayev et al., 2019).

The common ice plant, *Mesembryanthemum crystallinum*, is an intensively studied facultative CAM model species, in which C_3_ photosynthesis‐performing plants can switch to CAM in response to salinity, water‐deficit, low‐humidity, and abscisic acid treatments (Cushman & Borland, 2002; Taybi & Cushman, 1999). Moreover, the common ice plant provides a valuable system to compare the enzyme activity patterns (Holtum & Winter, 1982; Nosek et al., 2018; Winter et al., 1982) or mRNA expression profiles between the C_3_ and CAM photosynthesis states (Cushman et al., 2008; Toyokura et al., 2025). Utilizing model species as a source for CAM enzymatic and regulatory machinery to introduce CAM into C_3_ and C_4_ photosynthetic crops presents a promising strategy for improving WUE in plants (Borland et al., 2014; Lim et al., 2019; Lim et al., 2025; Schiller & Bräutigam, 2021; Yang et al., 2015; Yang et al., 2023).

*M. crystallinum* expresses multiple malic enzyme isoforms with distinct subcellular localizations. Biochemical fractionation studies have identified cytosolic NADP‐ME isoforms and mitochondrial NAD‐ME activity in this species (Winter et al., 1982). NAD(P)‐malic enzymes are critical enzymes involved in regulating malate metabolism, with two distinct types, NADP‐ME and NAD‐ME, each comprising multiple isoforms localized in various cellular organelles, where they perform specific functions (Chen et al., 2019; Sun et al., 2019). NADP‐ME plays a pivotal role in carbon metabolism, significantly contributing to plant photosynthesis by generating NADPH, which serves as a reducing agent for anabolic reactions (Sun et al., 2019). In a previous study, four *M. crystallinum* malic enzyme genes (*McNADP‐ME1*, *McNADP‐ME2*, *McNAD‐ME1*, and *McNAD‐ME2*) were overexpressed in *Arabidopsis thaliana* to assess their effects on stomatal conductance and titratable acidity. That study found that *McNADP‐ME1* and *McNADP‐ME2* overexpression led to the greatest decreases in stomatal conductance (Lim et al., 2019). Building upon these findings, the present study focuses specifically on cytosolic *McNADP‐ME1*, providing a comprehensive characterization of its effects on water‐deficit stress tolerance, including detailed analyses of acute and chronic water‐deficit stress responses, flavonoid accumulation, ROS‐scavenging capacity, and seed yield under water‐limiting conditions—aspects that were not examined in the earlier work. Similar results have been observed in other NADP‐ME overexpression studies. For example, overexpression of NADP‐ME from *Zea mays* reduced stomatal conductance and improved WUE in tobacco (Laporte et al., 2002). Overexpression of an NADP‐ME isoform in *A. thaliana* resulted in decreased malate and fumarate content (Fahnenstich et al., 2007). Ectopic expression of a maize NADP‐ME in guard and vascular companion cells of tobacco improved WUE while also driving earlier flowering and shortening of the plant life cycle (Müller et al., 2018). Lastly, overexpression of NADP‐ME resulted in increased photosynthetic efficiency, reduced leaf malate content, and superior performance under water‐deficit conditions in rice (Swain et al., 2021).

Flavonoids are a diverse class of plant secondary metabolites recognized for their non‐enzymatic antioxidant properties, playing key roles in scavenging ROS, providing UV protection, and enhancing pathogen defenses. NADP‐ME plays a critical role in supplying reducing equivalents in the form of NADPH, which is essential for the biosynthesis of flavonoids and lignin, which play critical roles in scavenging ROS (Chen et al., 2019; Sun et al., 2019). In addition to its biosynthetic role, NADPH is a vital factor in redox signaling and in the maintenance of cellular homeostasis (Scheibe, 2019). This coenzyme is required in the ascorbate‐glutathione pathway, which protects cells from oxidative damage, and is involved in the regulation of metabolic pathways via NADPH‐dependent thioredoxin reductases (NTRs) through thiol group reduction (Mittler et al., 2004). Furthermore, NADPH is essential for the activation of NADPH oxidases, known as respiratory burst oxidase homologs (RBOHs), which play a crucial role in cellular redox signaling (Chen et al., 2019; Mittler et al., 2022; Sun et al., 2019). NADP‐ME has also been implicated in various non‐photosynthetic functions including responses to fungal and bacterial pathogens (Casati et al., 1999; Mhamdi & Noctor, 2016; Singh et al., 2016; Voll et al., 2012), cold stress (Crecelius et al., 2003), salinity and osmotic stress (Badia et al., 2015; Cheng & Long, 2007; Guo et al., 2018; Liu et al., 2007), ozone stress, heavy metal stress, UV‐B radiation (Casati et al., 1999; Drincovich et al., 1998), and wounding responses, among other biotic and abiotic stresses (Casati et al., 1999; Chen et al., 2019; Doubnerová & Ryšlavá, 2011; Sun et al., 2019).

Based upon these previous results, we hypothesize that NADP‐ME overexpression might improve water‐deficit stress tolerance through multiple physiological pathways. Here, we demonstrate that the overexpression of *McNADP‐ME1* in *A. thaliana* decreased stomatal conductance and water loss through reductions in both stomatal density and stomatal aperture, while also increasing the accumulation of ROS‐scavenging flavonoids and the expression of key flavonoid biosynthetic genes, which in combination contribute to enhanced water‐deficit stress tolerance.

## RESULTS

### mRNA expression patterns of the malic enzyme gene family in ice plant

**Figure 1:**
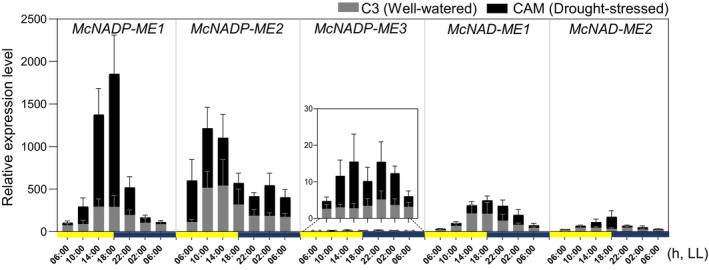
Gene expression analysis of the malic enzyme family in the facultative CAM species *M. crystallinum*. The relative transcript abundance of *McNADP‐ME1*, *McNADP‐ME2*, *McNADP‐ME3*, *McNAD‐ME1*, and *McNAD‐ME2* was analyzed over a 24‐h time course under well‐watered (gray bars) and water‐deficit‐stressed (black bars) conditions. Plants were grown under a 12 h light/12 h dark photoperiod, and on the sampling day, tissues were collected every 4 h from 06:00 to 06:00 the following day under continuous light (LL) conditions. Two‐month‐old plants were subjected to a two‐week water‐deficit stress treatment prior to sampling. Gene expression data were normalized to the housekeeping genes *McActin* and *McUBQ* and are presented as fold changes relative to the *McNADP‐ME3* well‐watered sample at 06:00. Values represent the mean ± SD (*n* = 6 with three biological replicates).

In response to water‐deficit or salinity stress, *M*. *crystallinum* can shift from C_3_ photosynthesis to CAM, a process that optimizes WUE by altering the timing of CO_2_ uptake. To identify the genes that facilitate this adaptive response, the mRNA expression patterns of NAD(P)‐ME genes were investigated using RT‐qPCR in plants performing either C_3_ photosynthesis or CAM induced by water‐deficit stress using samples collected in triplicate every 4 h for 24 h. *McNADP‐ME1* exhibited peak expression at dusk, whereas *McNADP‐ME2* showed the highest expression in mid‐morning. In contrast, *McNADP‐ME3* displayed the lowest expression among all other ME genes. The two mitochondrial NAD‐ME genes encoding the alpha and beta subunits (*McNAD‐ME1* and *McNAD‐ME2*), respectively, exhibited intermediate levels of expression that peaked at dusk (Figure 1). We concluded that *McNADP‐ME1*, which exhibited the highest mRNA expression among all five gene family members, likely serves as a key CAM enzyme, and was thus investigated further. Accordingly, *McNADP‐ME1* was expressed under the control of the *CaMV 35S* promoter with an sGFP tag fused to the C‐terminus. This C‐terminal fusion construct was then introduced into *A*. *thaliana* via *Agrobacterium*‐mediated transformation. Eight independent T_3_ transgenic lines were obtained from which three lines (#2, #3, and #7) with strong relative mRNA expression and sGFP expression were selected for further detailed analysis (Figure S1). Leaf epidermal cells were examined using confocal laser‐scanning microscopy to visualize sGFP alongside chloroplast autofluorescence, and the images were subsequently merged. The *35S::McNADP‐ME1*‐*sGFP* fusion protein localized to the cytosol consistent with its predicted subcellular localization using the FUEL‐mLoc subcellular localization prediction server (Wan et al., 2017). The *35S::sGFP* fusion construct (empty vector), which served as a control, showed GFP localization in both the cytosol and the nucleus (Figure S2).

### *McNADP‐ME1* exhibits malate decarboxylation activity

**Figure 2:**
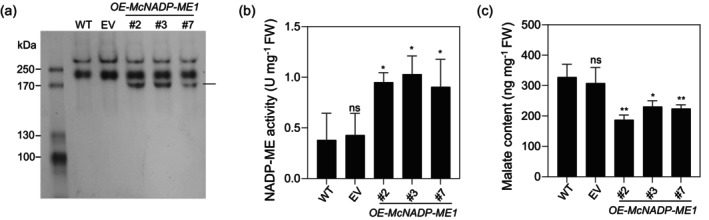
*McNADP‐ME1* overexpression increases NADP‐ME activity. (a) Native PAGE analysis of NADP‐ME activity in leaves of wild‐type (WT), *CaMV35S::sGFP* empty vector (EV) and three *McNADP‐ME1‐*overexpressing lines (#2, #3, and #7). The arrow indicates a unique band pattern specifically observed in *McNADP‐ME1‐*overexpressing lines. (b) Quantification of NADP‐ME activity (*n* = 3). (c) Quantification of malate content. Values represent means ± SD, ns = non‐significant, *P < 0.05 and **P < 0.01, one‐way ANOVA with Dunnett's multiple comparison test.

To confirm that *McNADP‐ME1* encodes an enzymatically active NADP‐ME enzyme, crude protein extracts from the wild‐type (WT), empty vector (EV) control line expressing only sGFP, and the three independent transgenic lines were assayed for NADP‐ME activity by native polyacrylamide gel electrophoresis followed by NADP‐ME activity staining. Two bands corresponding to ~400 kD and ~200 kD, respectively, were detected in all samples, indicating the presence of *A. thaliana* endogenous NADP‐ME isoforms (Figure 2a). By contrast, a lower molecular weight band (~150 kD) was only visible in the transgenic lines. Quantitative NADP‐ME activity was further determined spectrophotometrically and revealed much higher activity in all three transgenic lines compared with the WT (Figure 2b). Next, we measured malate content and found that the transgenic lines contained 0.6‐ to 0.7‐fold less malate compared with the WT and EV control lines (Figure 2c). This result suggests that the introduction of *McNADP‐ME1* in *A. thaliana* accelerated oxidative malate decarboxylation yielding pyruvate and NADPH while releasing CO_2_ into the cytosol.

### *McNADP‐ME1* overexpression decreases organ size and biomass under low‐light conditions

**Figure 3:**
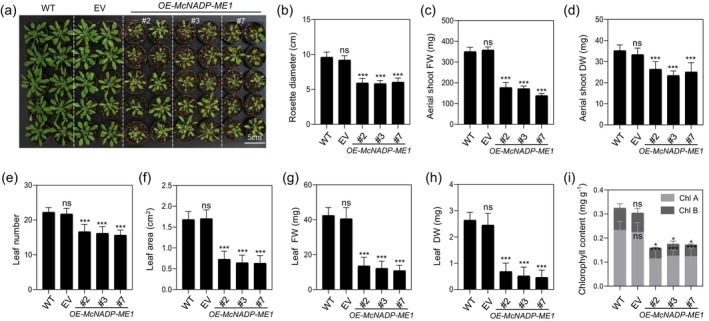
*McNADP‐ME1* overexpression decreases biomass in *Arabidopsis*. (a) Representative images of 28‐day‐old wild‐type (col‐0, WT), *CaMV35S::sGFP* empty vector (EV) control and three *McNADP‐ME1* overexpression lines (#2, #3 and #7). Scale bar, 5 cm. (b) Rosette diameter (*n* = 24 with three biological replicates). (c) Aerial shoot fresh weight (*n* = 24 with three biological replicates). (d) Aerial shoot dry weight (*n* = 24 with three biological replicates). (e) Leaf number (*n* = 24 with three biological replicates). (f) Leaf area (*n* = 24 with three biological replicates). (g) Leaf fresh weight (*n* = 24 with three biological replicates). (h) Leaf dry weight (*n* = 24 with three biological replicates). (i) Chlorophyll contents of 21‐day‐old WT, EV control line and three *McNADP‐ME1* overexpression lines (*n* = 6 with three biological replicates). Values represent means ± SD, ns = non‐significant, *P < 0.05, **P < 0.01, and ***P < 0.001, one‐way ANOVA with Dunnett's multiple comparison test.

Phenotypic analysis was conducted using three independent *A. thaliana* transgenic lines overexpressing *McNADP‐ME1* with sGFP under the control of the *CaMV::35S* promoter (*OE‐McNADP‐ME1‐sGFP* lines #2, #3, and #7), along with an EV control line (Figure 3a). Under soil‐grown conditions, *McNADP‐ME1* overexpression lines exhibited a 1.6‐fold reduction in rosette diameter (Figure 3b). Additionally, the fresh and dry weights of aerial shoots decreased by 2.0‐ to 2.5‐fold and 1.3‐ to 1.5‐fold, respectively (Figure 3c,d). Similarly, the number of leaves was reduced by 1.3‐ to 1.4‐fold (Figure 3e). When evaluating leaf area, the fourth leaf of *McNADP‐ME1* overexpression lines displayed a 2.3‐ to 2.7‐fold reduction compared to the control lines (Figure 3f). Likewise, the fresh and dry weights of the fourth leaf were 3.1‐ to 3.9‐fold and 3.8‐ to 5.7‐fold lower, respectively (Figure 3g,h). Chlorophyll a/b content was also reduced by 1.8‐ to 2.1‐fold (Figure 3i). These phenotypic analyses under 12‐hour light/12‐hour dark growth conditions indicated that organ size, biomass, and chlorophyll content were decreased, suggesting a reduction in photosynthetic activity.

### *McNADP‐ME1* overexpression decreases stomatal size and CO_2_ assimilation while increasing intrinsic water‐use efficiency

**Figure 4:**
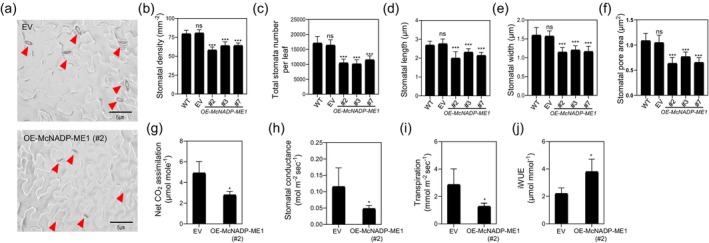
*McNADP‐ME1* overexpression improves water‐use efficiency (WUE) in *Arabidopsis thaliana*. (a) Representative images of stomatal aperture and density of the *CaMV35S::sGFP* (EV) control line and *McNADP‐ME1* overexpression line (#2). Red arrowheads indicate the position of stomata in the lower epidermis. (b) Stomatal density (*n* = 15 with three biological replicates). (c) Total stomatal number per leaf (*n* = 15 with three biological replicates). (d) Stomatal length (*n* = 30 with three biological replicates). (e) Stomatal width (*n* = 30 with three biological replicates). (f) Stomatal pore area (*n* = 30 with three biological replicates). (g) Net CO_2_ assimilation (*n* = 3). (h) Stomatal conductance (*n* = 3). (i) Transpiration (*n* = 3). (j) iWUE (*n* = 3). Values represent means ± SD, ns = non‐significant, *P < 0.05 and ***P < 0.001, one‐way ANOVA with Dunnett's multiple comparison test.

The stomatal density of *McNADP‐ME1* overexpression lines was reduced by approximately 1.2‐ to 1.4‐fold compared to the WT or EV plants (Figure 4a,b). To quantify the total number of stomata, images were captured over an area of 0.1 cm^2^ and the averaged results indicated that the number of stomata in *McNADP‐ME1* overexpression lines decreased by 1.5‐ to 1.7‐fold (Figure 4c). Furthermore, the stomatal width and length were reduced by 1.3‐ to 1.4‐fold and 1.2‐ to 1.3‐fold, respectively, leading to a 1.4‐ to 1.7‐fold decrease in stomatal area (Figure 4d–f). These observations suggest that *McNADP‐ME1* overexpression lines exhibited fewer and smaller stomata compared to the control lines. The observed reductions in stomatal density and size were likely contributing factors to the overall decrease in plant size associated with *McNADP‐ME1* overexpression.

The leaves of *McNADP‐ME1* overexpression plants exhibited lower stomatal density than control plants (Figure 4b–f). Changes in stomatal density can influence various physiological processes, including net CO_2_ assimilation, transpiration, stomatal conductance, and instantaneous water‐use efficiency (iWUE). Therefore, gas‐exchange measurements were conducted to compare the control and *McNADP‐ME1* overexpression plants. The results showed that net CO_2_ assimilation decreased by 1.8‐fold, stomatal conductance decreased by 2.4‐fold, and transpiration decreased by 2.2‐fold (Figure 4g–i). Conversely, iWUE increased by 1.7‐fold in the *McNADP‐ME1* overexpression lines indicating improved WUE (Figure 4j).

### *McNADP‐ME1* overexpression decreases size of reproductive structures and seed yield

**Figure 5:**
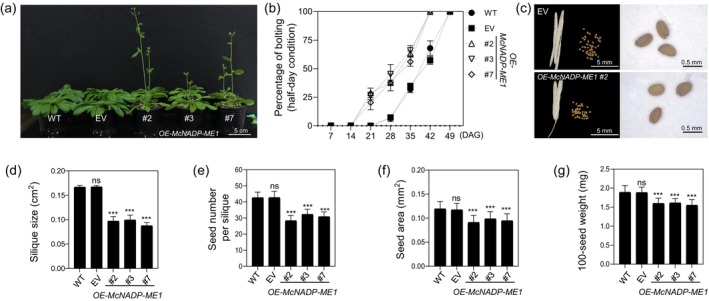
*McNADP‐ME1* overexpression accelerates flowering time and decreases seed yield. (a) Representative image of 28‐day‐old wild‐type (WT), *CaMV35S::sGFP* empty vector (EV) control, and three *McNADP‐ME1‐*overexpressing lines (#2, #3, and #7). Seeds were germinated and grown in soil for 4 weeks under a 12‐h photoperiod. (b) Quantification of flowering time (*n* = 3 biological replicates with 36 plants per replicate). (c) Representative images of seed number per dried silique and seed of the EV control and *McNADP‐ME1*‐overexpressing line (#2). (d) Silique area (*n* = 15). (e) Seed number per silique (*n* = 30). (f) Seed area (*n* = 90). (g) 100‐seed weight (*n* = 15). Values represent means ± SD, ns = non‐significant, and ***P < 0.001, two‐way ANOVA with Dunnett's multiple comparison test.

Over time, *McNADP‐ME1* overexpression lines showed an accelerated transition to the reproductive phase. *McNADP‐ME1* overexpression lines displayed bolting approximately 2 weeks earlier than the control lines, indicating a more rapid shift to the reproductive stage (Figure 5a,b). Associated with the accelerated developmental timing, the size of the reproductive structures and seed yield decreased. The silique size was reduced by 1.7‐ to 1.9‐fold in the *McNADP‐ME1* overexpression lines compared to the control lines (Figure 5c,d). Additionally, the number of seeds per silique was decreased 1.3‐ to 1.5‐fold in the *McNADP‐ME1* overexpression lines, with seed area reduced by 1.2‐ to 1.3‐fold (Figure 5e,f). The 100‐seed weight was also reduced by 1.2‐fold in the *McNADP‐ME1* overexpression lines (Figure 5g).

### *McNADP‐ME1* overexpression attenuates water‐deficit stress

**Figure 6:**
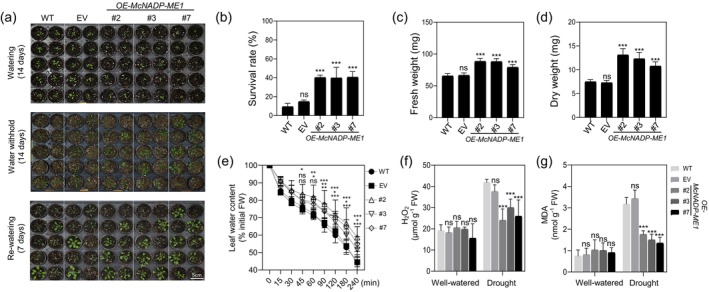
*McNADP‐ME1* overexpression improves water‐deficit stress tolerance under acute water‐deficit stress conditions. (a) Representative images of three *McNADP‐ME1* overexpressing lines (#2, #3, and #7), wild‐type (col‐0, WT), and *CaMV35S::sGFP* empty vector (EV) control line at 14, 28, and 35 days after germination. Water irrigation was stopped at 14 days after germination, withheld for 14 days and rewatered for 7 days. (b) Survival rate (*n* = 3 replicates with 36 plants per replicate). (c) Fresh weight (*n* = 24 with three biological replicates). (d) Dry weight (*n* = 24 with three biological replicates). (e) Quantification of leaf water content. Three *McNADP‐ME1* overexpressing lines (#2, #3, and #7), Col‐0 wild type (WT) and the *CaMV35S::sGFP* empty vector (EV) control line after detached leaves were exposed to air under room temperature conditions. (f) H_2_O_2_ content (*n* = 5). (g) Malondialdehyde (MDA) content (*n* = 5). Values represent means ± SD, ns = non‐significant, *P < 0.05, **P < 0.01, and ***P < 0.001, one‐way ANOVA with Dunnett's multiple comparison test.

To assess the alleviation of acute water‐deficit stress in the *McNADP‐ME1* overexpression lines, plants were grown for 21 days, followed by a two‐week period without irrigation and a subsequent seven‐day recovery period with re‐irrigation (Figure 6a). Under these conditions, only 9.4%–14.8% of the control lines survived, whereas the *McNADP‐ME1* overexpression lines showed a survival rate of 39.8%–40.7% (Figure 6b; Figure S3). Under water‐deficit conditions, leaf fresh weight and dry weight were 1.2‐ to 1.3‐fold and 1.4‐ to 1.8‐fold higher, respectively, in the *McNADP‐ME1* overexpression lines compared to the control lines (Figure 6c,d). The enhanced survival was further validated through leaf air‐drying experiments, which demonstrated that *McNADP‐ME1* overexpression reduced the rate of water loss. The *McNADP‐ME1* overexpression line (#2) exhibited a slower reduction in leaf water content than in the control lines starting at 45 min. From 90 min onwards, all overexpression lines showed statistically significant differences compared to the control lines (Figure 6e). Additionally, H_2_O_2_ and MDA levels, indicators of oxidative stress, were lower in the *McNADP‐ME1* overexpression lines, confirming reduced water‐deficit stress‐related effects (Figure 6f–g).

**Figure 7:**
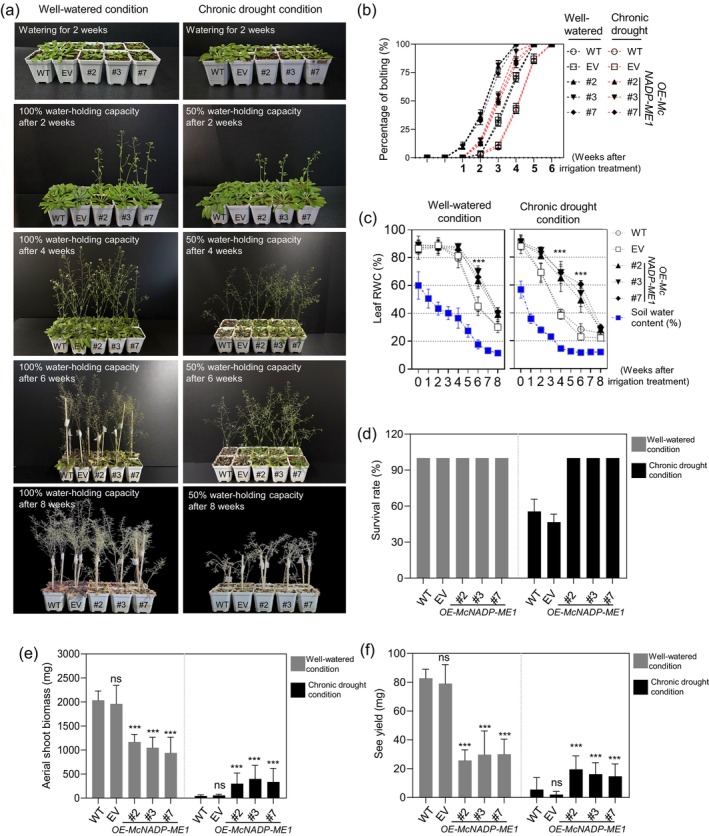
*McNADP‐ME1* overexpression improves plant biomass and seed yield under chronic water‐deficit stress conditions. (a) Representative images of Col‐0 wild type (WT), *CaMV35S::sGFP* empty vector (EV) control lines and *McNADP‐ME1* overexpression lines (#2, #3 and #7) at 2, 4, 6, and 8 weeks under well‐watered (100% soil water‐holding capacity) and chronic water‐deficit stress (50% soil water‐holding capacity) conditions. Two‐week‐old well‐watered plants were grown for 8 weeks under different soil water‐holding capacities (100% or 50%). (b) Quantification of bolting time (*n* = 3 replicates with 36 plants per replicate). (c) Quantification of relative water content (RWC) of leaf under different conditions of soil water‐holding capacity (*n* = 3 replicates with 12 detached leaves per replicate). (d) Quantification of plant survival rate (*n* = 3 replicates with 36 plants per replicate). (e) Quantification of dry weight of aerial tissue per plant (*n* = 15). (f) Quantification of seed weight per plant (*n* = 15). Values represent means ± SD, ns = non‐significant, and ***P < 0.001, one‐way ANOVA with Dunnett's multiple comparison test.

To evaluate the long‐term effects of *McNADP‐ME1* overexpression, plants were subjected to chronic water‐deficit stress conditions by maintaining soil water‐holding capacity (SWC) at 100% (well‐watered condition) or 50% (chronic water‐deficit stress condition) for 8 weeks (Figure 7a). Under the chronic water‐deficit stress, the time to bolting was delayed by about 1 week for the WT, EV, and *McNADP‐ME1* overexpression lines compared to the well‐watered condition. However, the overexpression lines (#2, #3, and #7) still flowered significantly earlier than the control lines (Figure 7b). This result shows that the early flowering trait of the *McNADP‐ME1* overexpression lines is maintained even when growth is limited by water stress. The *McNADP‐ME1* overexpression lines also showed better water status during the long‐term water‐deficit conditions. Early in the treatment, no significant differences in leaf relative water content (RWC) were evident between the control and overexpression lines under both conditions (Figure 7c). However, under the well‐watered condition, the *McNADP‐ME1* overexpression lines maintained significantly higher RWC levels than the WT and EV control lines at 6 weeks of irrigation. Under the chronic water‐deficit stress condition, the differences in leaf RWC appeared earlier, beginning at 4 weeks. By 6 weeks of chronic water‐deficit stress, the leaf RWC of the WT and EV control lines had dropped to an average of approximately 24.9%, while the three *McNADP‐ME1* overexpression lines maintained a much higher average RWC of about 55.6% (Figure 7c). The survival rates of the WT and EV control lines fell to between 45.5% and 46.6% under the chronic water‐deficit stress condition (Figure 7d). In contrast, all three *McNADP‐ME1* overexpression lines showed a 100% survival rate. Lastly, *McNADP‐ME1* overexpression led to better growth and higher yield under chronic water‐deficit stress. The dry weight of aerial tissues was significantly higher in the *McNADP‐ME1* overexpression lines than in the control lines (Figure 7e). Similarly, the total seed weight per plant was also increased under the chronic water‐deficit stress condition (Figure 7f). Compared to the WT, the *McNADP‐ME1* overexpression lines #2, #3, and #7 showed 3.2‐fold, 2.9‐fold, and 2.6‐fold higher seed yields, respectively. These results demonstrate that *McNADP‐ME1* overexpression improves both plant biomass and seed yield during long‐term water‐deficit conditions.

Detailed stomatal measurements taken after 4 weeks of water limitation showed that the *McNADP‐ME1* overexpression lines maintained smaller stomatal length and width than WT and EV control lines under both well‐watered and water‐deficit conditions (Figure S4a–c). Accordingly, the stomatal pore area was smaller in the *McNADP‐ME1* overexpression lines relative to the control lines under well‐watered conditions (Figure S4d). However, under chronic water‐deficit stress conditions, the stomatal pore area was significantly larger in the *McNADP‐ME1* overexpression lines compared with the control lines, likely because the control lines experienced more severe water‐deficit stress‐induced stomatal closure (Figure S4d).

Next, we performed detailed gas‐exchange analyses on the plants subjected to chronic water‐deficit stress. Under well‐watered conditions, the *McNADP‐ME1* overexpression lines exhibited reduced net CO_2_ assimilation, stomatal conductance, and transpiration rates relative to the control lines (Figure S5a–c) as well as greater iWUE (Figure S5d). However, under chronic water‐deficit stress conditions, the *McNADP‐ME1* overexpression lines showed greater net CO_2_ assimilation, stomatal conductance, and transpiration rates relative to the control lines (Figure S5a–c), consistent with the greater stomatal pore areas observed under chronic water‐deficit stress conditions. Interestingly, the *McNADP‐ME1* overexpression lines also showed even greater iWUE under the chronic water‐deficit stress conditions than those observed under well‐watered conditions, consistent with the greater leaf RWC, survival rates, aerial shoot biomass, and seed yield seen under the chronic stress conditions (Figure 7).

### *McNADP‐ME1* overexpression improves growth under high‐light conditions

*McNADP‐ME1*‐overexpressing plants exhibited improved growth under high‐light stress (350 μmol m^−2^ s^−1^) conditions compared to the control lines. In contrast, *A. thaliana* growth was reduced compared with control lines under low‐light conditions (100 μmol m^−2^ s^−1^) (Figure 3), *McNADP‐ME1*‐overexpressing lines showed improved leaf number, rosette diameter, leaf fresh weight, and leaf dry weight when grown under high‐light conditions (Figure S6). The improved performance under both water‐deficit and high‐light stress suggested that *McNADP‐ME1* overexpression might enhance protective mechanisms against ROS‐mediated damage. Flavonoids are well‐characterized stress‐responsive metabolites that function as antioxidants under both water‐deficit and high‐light conditions (Agati et al., 2012; Nakabayashi et al., 2014). Because NADP‐malic enzyme activity generates NADPH and provides carbon precursors that can support the phenylpropanoid pathway (Casati et al., 1999; Doubnerová & Ryšlavá, 2011), we hypothesized that the higher *McNADP‐ME1* activity in transgenic lines might enhance flavonoid biosynthesis. To test this hypothesis, we measured total flavonoid content and analyzed the expression of key flavonoid biosynthetic genes in *McNADP‐ME1*‐overexpressing plants under both well‐watered and water‐deficit stress conditions.

### *McNADP‐ME1* overexpression increases total flavonoid content by modulating the flavonoid synthesis pathway

**Figure 8:**
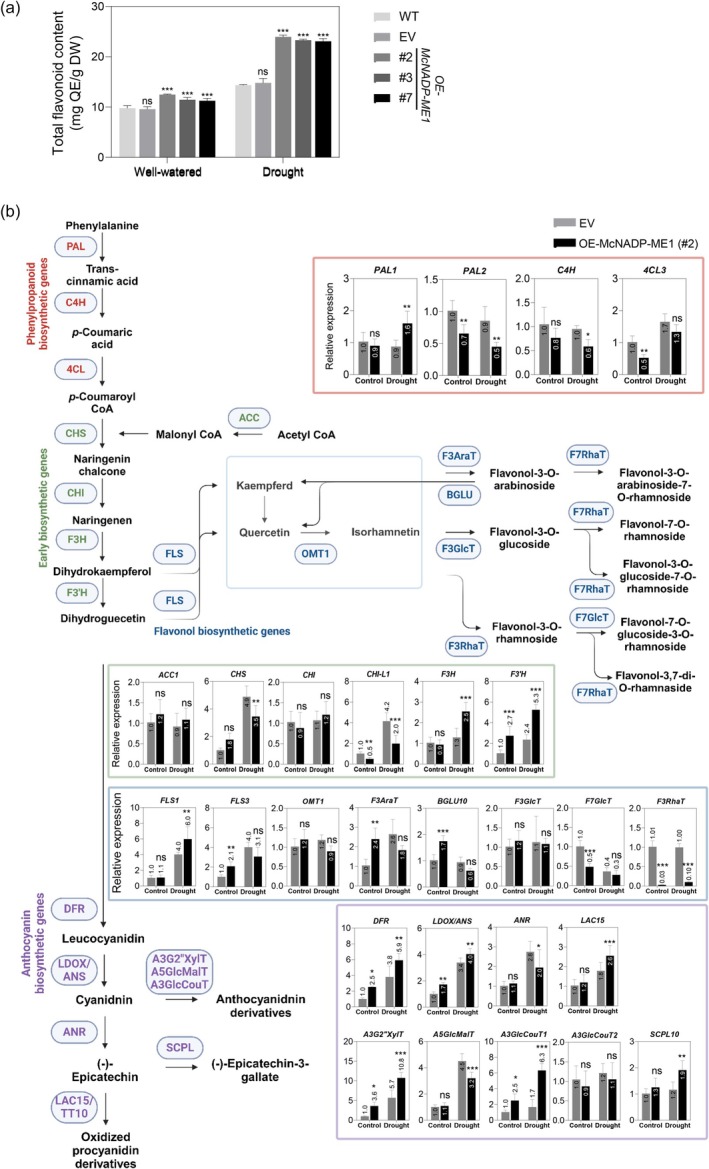
*McNADP‐ME1* overexpression increases total flavonoid content and alters expression levels of flavonoid biosynthetic genes. (a) Total flavonoid content (*n* = 6 with three biological replicates). (b) General biosynthetic pathway of flavonoid. 4CL, 4‐coumaric acid: CoA ligase; A3G2″XylT, Anthocyanin 3‐O‐glucoside: 2″‐O‐xylosyltransferase; A3GlcCouT, Anthocyanin coumaroyltransferase; A5GlcMalT, Anthocyanin malonyltransferase; ACC, acetyl‐CoA carboxylase; ANR, anthocyanidin reductase; ANS, anthocyanidin synthase; BGLU, Anthocyanin 3‐O‐6″‐O‐coumaroyl glucoside: glucosyltransferase; C4H, cinnamic acid 4‐hydroxylase; CHI, chalcone isomerase; CHS, chalcone synthase; DFR, dihydroflavonol 4‐reductase; F3′H, flavonoid 3′‐hydroxylase; F3AraT, Flavonol 3‐O‐arabinosyltransferase; F3GlcT, Flavonoid 3‐O‐glucosyltransferase; F3H, flavanone 3‐hydroxylase; F3RhaT, Flavonol 3‐O‐rhamnosyltransferase; F7GlcT, Flavonol 7‐O‐glucosyltransferase; F7RhaT, Flavonol 7‐O‐rhamnosyltransferase; FLS, flavonol synthase; LAC15, Laccase; LDOX, Leucoanthocyanidin dioxygenase; OMT1, O‐methyltransferase 1; PAL, phenylalanine ammonia lyase; SCPL, Anthocyanin sinapoyltransferase.

We measured total flavonoid content and found that following water‐deficit stress, the *McNADP‐ME1* overexpression lines exhibited a 1.6‐ to 1.7‐fold increase in total flavonoid content compared to control lines (Figure 8a). Quantitative real‐time PCR (RT‐qPCR) was conducted to analyze the expression levels of flavonoid biosynthesis‐related genes, including PAL, C4H, 4CL, ACC, CHS, CHI, F3H, F3′H, FLS, OMT1, F3AraT, F7RhaT, BGLU, F3GlcT, F7GlcT, DFR, LDOX, ANS, ANR, LAC15, A3G2″XylT, A5GlcMalT, A3GlcCouT, and SCPL, in *A. thaliana* subjected to water‐deficit stress. Under well‐watered conditions, the *McNADP‐ME1* overexpression line exhibited higher steady‐state transcript abundance of F3′H, FLS3, F3AraT, BGLU10, DFR, LDOX/ANS, A3G2″XylT, and A3GlcCouT1 compared to the control lines (Figure 8b). In contrast, the expression levels of PAL2, 4CL3, CHI‐L1, F7GlcT, and F3RhaT were lower in the *McNADP‐ME1* overexpression lines than in the control lines. Under water‐deficit stress conditions, the expression of PAL1, F3H, F3′H, FLS1, DFR, LDOX/ANS, LAC15, A3G2″XylT, A3GlcCouT1, and SCPL10 showed significant increases in relative transcript abundance in the *McNADP‐ME1* overexpression lines compared to the control lines. Conversely, the transcript abundance of PAL2, C4H, CHS, CHI‐L1, F3RhaT, F7RhaT, ANR, and A5GlcMalT was lower in the *McNADP‐ME1* overexpression lines than in the control lines under water‐deficit stress conditions (Figure 8b).

## DISCUSSION

We have exploited malic acid decarboxylation, a key step in the CAM pathway, to enhance the water‐deficit stress tolerance of *A. thaliana* through the overexpression of NADP‐ME from *M. crystallinum* (Lim et al., 2019; Lim et al., 2025). We confirmed that *McNADP‐ME1* overexpression reduced stomatal conductance, which not only improved water‐use efficiency, but also enhanced the production of reactive oxygen species (ROS) scavenging flavonoids, further contributing to improved abiotic stress tolerance. *M. crystallinum*, a facultative CAM species, serves as an excellent model for identifying C_4_ metabolism genes involved in the core CAM diel cycle, based upon their mRNA expression patterns in response to salinity or water‐deficit stress (Cushman et al., 2008; Lim et al., 2019). We showed that among the *McNAD(P)‐ME* genes, *McNADP‐ME1* exhibited the highest mRNA abundance among all five genes, with peak expression at 18:00 suggesting that *McNADP‐ME1* accumulation occurs in anticipation of the daytime decarboxylation phase of CAM. However, because we used a strong, constitutive promoter to drive the expression of *McNADP‐ME1* in the current study, we do not expect a CAM‐like cycle to manifest itself as this would require the use of a promoter that would provide a temporal expression pattern like that observed in *M. crystallinum* (Figure 1). We confirmed that *NADP‐ME1* is localized specifically in the cytosol. This result is consistent with previous studies that have reported that NADP‐ME isoforms are located in various subcellular compartments including the cytosol (Lim et al., 2019; Maier et al., 2011; Tronconi et al., 2008; Wheeler et al., 2005).

### *McNADP‐ME1*‐mediated stomatal regulation, improves water‐use efficiency and mitigates growth penalties under high‐light conditions

*McNADP‐ME1* overexpression resulted in a reduction in stomatal density, size, and conductance, leading to significantly enhanced WUE (Figure 4). These modifications dramatically improved survival rates and slowed the rate of leaf water loss following the imposition of water‐deficit stress (Figures 6 and 7; Figures [Link], [Link], [Link]). Previous studies have demonstrated that such *NADP‐ME* overexpression enhances WUE and drought tolerance while reducing soil water loss (Laporte et al., 2002; Müller et al., 2018). Similar results have been observed when stomatal density is limited by genetic manipulation of signaling components such as EPF and EPFL gene family members (Bertolino et al., 2019; Gray & Dunn, 2024; Hunt & Gray, 2009; Lu et al., 2019), where reduced density enhances soil water conservation and WUE while maintaining relatively normal growth under specific conditions (Caine et al., 2019; Hughes et al., 2017; Hunt & Gray, 2009; Mohammed et al., 2019). In our study, the observed reductions in stomatal aperture are likely due to decreased malate content (Figure 2), which plays a key role in stomatal movement (Mimata et al., 2022; Sasaki et al., 2022; Yang et al., 2025). Furthermore, malic acid decarboxylation by *McNADP‐ME1* elevates intracellular CO_2_ levels within guard cells, driving stomatal closure as CO_2_ is a key regulator of stomatal behavior (Engineer et al., 2016; Foyer et al., 2025; Tian et al., 2015).

Crucially, this *McNADP‐ME1*‐mediated regulation mitigates growth penalties specifically under higher light intensities. While reduced stomatal conductance often limits CO_2_ assimilation under standard growth conditions (100 μmol m^−2^ s^−1^ PAR) (Figure 4), consistent with reports in other *NADP‐ME* over‐expressors (Badia et al., 2015; Zell et al., 2010), these effects are overcome when plants are grown under high‐light conditions of 350 μmol m^−2^ s^−1^ PAR (Figure S6). This recovery might be driven by the internal CO_2_ fertilization effect. The internal CO_2_ generated by *McNADP‐ME1* decarboxylation acts as a localized carbon source for the CBB cycle (Müller et al., 2018; Oitaven et al., 2024). Under higher light intensity, the increased supply of ATP and NADPH allows the photosynthetic apparatus to fully utilize this internal CO_2_, thereby bypassing the stomatal limitation that typically hinders growth under lower light. Additionally, higher light often increases the vapor pressure deficit (VPD) and leaf temperature, which can cause significant stress in plants. The enhanced WUE and robust ROS‐scavenging capacity (Figures 6, 7, 8) of *McNADP‐ME1* overexpression provides superior protection against such environmental demands, shifting the balance from a growth penalty to a competitive advantage under high irradiance. Elevated CO_2_ is known to enhance stomatal responses to osmotic stress in *A. thaliana* (Leymarie et al., 1999), and our results suggest that *McNADP‐ME1* integrates these metabolic and physiological signals to optimize plant performance under high‐energy and water‐limited environments.

### McNADP‐ME1 overexpression accelerates flowering time

In addition to improving WUE and water‐deficit stress tolerance, we observed that NADP‐ME overexpressing lines displayed a reduced time to flowering of about 7 days (Figure 5). Such an acceleration in ontogeny was also observed in NADP‐ME overexpressing tobacco lines, which flowered and completed their life cycle 4–5 weeks faster than wild‐type controls (Müller et al., 2018). We also confirmed this early flowering phenotype under chronic water‐deficit stress (Figure 7). Under this condition, all lines, including the *McNADP‐ME1* overexpression lines, showed a flowering delay of about 1 week compared to well‐watered conditions (Figure 7b). This type of flowering delay is commonly seen in *A. thaliana* under water deficit and is often linked to the activation of floral repressors (Vatov & Gechev, 2025). However, even with this delay, the *McNADP‐ME1* overexpression lines still flowered significantly earlier than the control lines (Figure 7b).

The biochemical or physiological basis of the observed acceleration in flowering time is currently unknown. Studies examining the effects of elevated CO_2_ on flowering time vary widely with some studies showing no effect; others show either accelerated flowering time presumably through enhanced growth rates and more rapid leaf production or show delayed flowering time (Song et al., 2009; Springer & Ward, 2007; Ward et al., 2012). In the current study, enhanced intracellular CO_2_ concentrations due to malic acid decarboxylation likely accelerated flowering time consistent with previous observations in *A. thaliana* and other species (Tun et al., 2021). However, understanding the genetic or physiological basis of this acceleration will require further investigation.

### Contrasting stress tolerance outcomes of cytosolic *McNADP‐ME1* and *AtNADP‐ME2* overexpression

While our study demonstrates that constitutive overexpression of the *McNADP‐ME1* enhances water‐deficit stress tolerance in *A. thaliana*, a previous report showed that constitutive overexpression of the endogenous *Arabidopsis AtNADP‐ME2* isoform resulted in reduced tolerance to osmotic stress agents such as mannitol and polyethylene glycol (PEG). Both studies employed the CaMV 35S constitutive promoter and both enzymes were localized to the cytosol, yet the physiological outcomes were distinctly different. Several factors might contribute to this striking contrast. First, *McNADP‐ME1* originates from *M. crystallinum*, a facultative CAM species where NADP‐ME plays a central role in nocturnal malate decarboxylation and stress‐induced metabolic shifts (Figure 1). In contrast, *AtNADP‐ME2* is a native *Arabidopsis* gene that is constitutively expressed at high levels in all tissues under normal growth conditions and shows no demonstrated abiotic stress‐induced gene function (Wheeler et al., 2005). Second, *AtNADP‐ME2* exhibited the highest specific activity among all *Arabidopsis* NADP‐ME isoforms. Thus, constitutive overexpression of this gene might drive excessive cytosolic NADPH production and perturb primary metabolism, which might explain the reduced accumulation of osmoprotectants and increased sensitivity to osmotic stress (Badia et al., 2015). In comparison, *McNADP‐ME1* activity appears more moderate and might be more effectively coupled to adaptive pathways such as stomatal regulation via internal CO_2_ elevation and enhanced flavonoid biosynthesis for ROS scavenging (Figures 4, 7, 8). Third, although both enzymes are cytosolic, the metabolic outcomes of their overexpression differ substantially. Our results suggest that *McNADP‐ME1* integrates stress‐responsive signaling networks involving stomatal CO_2_ sensing and secondary metabolism, whereas *AtNADP‐ME2* overexpression primarily amplifies basal metabolic flux without activating these protective mechanisms. However, the precise molecular basis for these differences remains to be fully elucidated. These findings underscore the importance of enzyme origin, biochemical properties, and metabolic context when engineering stress tolerance using NADP‐ME genes. The CAM‐derived *McNADP‐ME1* appears to confer a unique advantage by linking carbon metabolism with water conservation and antioxidant defense, a feature not observed with the endogenous *Arabidopsis* isoform.

### The role of NADP‐ME in enhancing antioxidant defense and stress tolerance through flavonoid biosynthesis

To confirm the hypothesis that the increased ROS‐scavenging activity observed in our NADP‐ME lines (Figure 6f,g) was related to enhanced flavonoid accumulation, we showed that the total flavonoid content of the *McNADP‐ME1* overexpression lines was significantly increased under both well‐watered and water‐deficit stress conditions compared to control lines (Figure 8a). Furthermore, we demonstrated that *McNADP‐ME1* overexpression enhanced the mRNA expression of key enzymes of the early committed steps of the flavonoid pathway including phenylalanine ammonium lyase (APL), chalcone synthase (CHS), flavanone 3‐hydroxylase (F3H), and flavonoid 3′‐hydroxylase (F3′H) (Liu et al., 2021; Saito et al., 2013; Tohge et al., 2017). Several of the early steps of the flavonol biosynthetic pathway also showed significant increases in mRNA expression in the *McNADP‐ME1* lines under either well‐watered or water‐deficit stress conditions. Lastly, a majority of the genes encoding enzymes involved in the anthocyanin biosynthetic pathway showed increased mRNA expression in the *McNADP‐ME1* overexpression lines (Figure 8b). Anthocyanins are efficient ROS scavengers and thus are expected to play an important role in ameliorating damage under abiotic stress conditions while also maintaining their roles in cell signaling and plant development (Crecelius et al., 2003). These results confirm that flavonoids contribute to survival under water‐deficit stress conditions by strengthening the plant's antioxidant defense mechanisms. However, additional experiments are needed to address the possible roles of ROS‐scavenging enzymes and compounds not associated with the flavonoid pathway.

## CONCLUSION

Here, we show that constitutive overexpression in *A. thaliana* of the most abundantly expressed, cytosolic‐localized NADP‐ME (*McNADP‐ME1*) from the facultative CAM plant, *M. crystallinum*, resulted in a decrease in vegetative biomass and seed yield, but afforded greater WUE arising from reduced stomatal density, conductance and size, net CO_2_ assimilation, and transpiration. However, *McNADP‐ME1* overexpression significantly improved water‐deficit stress tolerance under both acute and chronic stress conditions and significantly enhanced biomass and seed yield under these conditions compared with control plants. In addition to slowing the rate of plant water loss and increasing iWUE, under chronic water‐deficit stress conditions *McNADP‐ME1*‐overexpressing plants displayed improved CO_2_ assimilation rates, stomatal conductance, and transpiration rates compared to control plants. These results indicate that engineering strategies involving *McNADP‐ME1* might be useful for preserving crop yields under field conditions where chronic hot and dry conditions are expected. In addition to improved WUE, our results showed that *McNADPME1* overexpression reduced ROS accumulation and lipid peroxidation and increased flavonoid content and mRNA expression of many flavonoid biosynthetic genes under water‐deficit stress conditions compared with control plants. In summary, *McNADPME1* plays a dual adaptive role in mitigating water‐deficit stress damage through not only improved water‐use efficiency, but also the production of ROS‐scavenging flavonoids, further improving stress tolerance. Lastly, we demonstrated that *McNADPME1* overexpression resulted in an acceleration in flowering time under both well‐watered and acute/chronic water‐deficit‐stress conditions. Acceleration of flowering time might be advantageous in regions with short growing seasons or when soil moisture loss is hastened by heat or drought.

## METHODS

### Plant material

Wild‐type (WT) *A. thaliana* (Col‐0), empty vector control (*CaMV35S::sGFP*), and three independent *McNADP‐ME1*‐overexpressing lines (*35S::McNADP‐ME1‐sGFP* #2, #3, and #7) were used in this study.

### RT‐qPCR of NAD‐ or NADP‐dependent malic enzyme genes in common ice plant

Wild‐type common ice plant (*M. crystallinum* L.) was grown in a growth chamber (HanKuk Scientific Instrument Company, HK‐GC800) modified for high‐light conditions under 12 h/12 h (light, 350 μmol m^−2^ s^−1^/dark) cycles at 22°C/18°C (day/night). After 8 weeks of growth, plants were divided into two groups. One group was maintained under well‐watered conditions with regular irrigation to sustain adequate soil moisture, whereas irrigation was completely withheld from the other group for 2 weeks to impose acute water‐deficit stress. Both well‐watered and water‐deficit stress‐treated plants were sampled on the same day. Leaf samples were collected over a 24‐h period beginning at 6:00 AM continuing at 4‐h intervals until 6:00 AM the following day. On the sampling day, plants were maintained under continuous light at 350 μmol m^−2^ s^−1^. At each time point, the fourth fully expanded leaf was harvested from four individual plants grown in separate pots to generate biological replicates. Three independent biological replicates were collected per treatment at each time point. Each plant was sampled only once to avoid potential effects of wounding stress.

Total RNA was isolated using the Apure™ Plant SFGR RNA Kit (APBIO, CAT. H4005). Purified RNA samples were processed into single‐stranded cDNA using oligo (dT) primers according to the RevertAid First Strand cDNA Synthesis Kit (Thermo Fisher Scientific, K1622). Quantitative real‐time PCR (RT‐qPCR) was performed using the Power SYBR Green PCR Master Mix (Thermo Fisher Scientific, CAT. 4367659) and subjected to the following cycle: 95°C for 10 min, and 40 cycles of 95°C for 10 sec, 60°C for 15 sec. RT‐qPCR was performed using the CFX Opus 96 Real‐Time PCR System (BIO‐RAD, CAT. 12011319). The housekeeping genes *McActin* (Cosentino et al., 2010) and *McUBQ* (Nosek et al., 2020) were used for normalization. The method for calculating using two housekeeping genes was previously described (Pfaffl, 2006).

### *Agrobacterium* transformation

The recombinant plasmids of the EV control (*CaMV 35S::sGFP*) and *CaMV35S::McNADP‐ME1‐sGFP* were chemically transformed into the *Agrobacterium tumefaciens* strain GV3101 for floral dipping of *A. thaliana* (ecotype Col‐0) (Zhang et al., 2006). T_0_ seeds were collected and screened on ½ strength MS basal medium supplemented with Gamborg Vitamins (pH 5.7), 10 g/L sucrose, 50 mg/L kanamycin, and 7 g/L Phyto agar in a growth chamber (HanKuk Scientific Instrument Company, HK‐GC800) under a 16‐h light/8‐h dark cycle for 10 days at 23°C during the day and 21°C at night, with a light intensity of 100 μmol m^−2^ s^−1^. To ensure consistency in seed quality, T_2_ homozygous seeds were harvested concurrently. A total of eight independent T_3_ transgenic lines were established and initially screened for *McNADP‐ME1* overexpression using semi‐quantitative PCR (Figure S1a). From this initial screening, the three lines showing the highest expression levels were selected for further analysis. Subsequent RT‐qPCR using *TIP41‐like* (AT4G34270) as an internal standard confirmed that these three independent T_3_ lines (#2A, #3B, and #7A) exhibited comparable and significantly elevated transcript abundance (Figure S1b). For simplicity and clarity in data presentation, these selected lines (#2A, #3B, and #7A) are referred to as #2, #3, and #7, respectively, throughout all figures. These validated lines were then utilized for subcellular localization and detailed physiological and phenotypic analyses.

### Subcellular localization

T_3_ transgenic seedlings of EV control (CaMV35S::sGFP), and *CaMV35S::McNAD‐ME1‐sGFP* were grown on ½ MS basal medium containing Gamborg Vitamins (pH = 5.7), 10 g/L sucrose, and 7 g/L Phyto agar in a Percival Scientific Model CU‐32 L growth chamber under a 16‐h photoperiod for 7 days. Leaf epidermal cells were observed using confocal laser‐scanning microscopy (FluoView FV1000, Olympus, Tokyo, Japan). GFP and chloroplast autofluorescence were excited at 488 nm with a laser and emission was collected at 510–560 nm and 680–700 nm, respectively. Subcellular localization predictions were performed using the FUEL‐mLoc subcellular localization prediction server at: http://bioinfo.eie.polyu.edu.hk/FUEL‐mLoc/citations.html (Wan et al., 2017). This prediction method uses essential GO terms to predict subcellular localizations, allows for multiple subcellular localizations for a protein from many different organisms, and is superior to other prediction programs that use sorting signals and PROSITE patterns.

### Plant growth conditions

For phenotypic quantification of vegetative rosette diameter, leaf area, and leaf fresh weight, seeds of each transgenic line were stratified in water at 4°C for 3 days and were sown in soil (Hanwol Bio), in square plastic pots (SIMPOL, Square Slit Planter 1, 6.9 cm square, 7.5 cm high) in a growth chamber (HanKuk Scientific Instrument Company, HK‐GC800), under a 12/12 (light, 100 μmol m^−2^ s^−1^/dark) photoperiod at 23°C/21°C (day/night). Four‐week‐old rosettes and detached leaves were photographed to measure rosette diameter and the area of the fourth true leaves. The fresh weight of the detached leaves was measured directly by gravimetric weighing. Rosette diameter and leaf area were quantified using ImageJ software.

### Enzyme extraction

Tissues of 8‐week‐old controls and *McNADP‐ME1* overexpressing lines were ground in liquid nitrogen and the resulting powder was suspended in 100 mM Tris–HCl, pH 7.5, 5 mM MgCl_2_, 2 mM EDTA, 10% (v/v) glycerol, and 10 mM 2‐mercaptoethanol in the presence of a protease inhibitor cocktail (Sigma) as described (Wheeler et al., 2005).

### *In vitro* NADP activity

NADP‐ME activity was determined spectrophotometrically using a standard reaction mixture composed of 50 mM Tris‐HCI (pH 7.5), 10 mM MgCl_2_, 0.5 mM NADP, and 10 mM L‐malate, in a final volume of 0.5 mL as described (Wheeler et al., 2005). The reaction was initiated by the addition of L‐malate and initial velocity studies were conducted by varying the concentration of one substrate around its Km value while keeping the other substrates at saturating levels. Kinetic parameters were determined from at least three independent measurements and fitted using nonlinear regression analysis based on the free concentrations of all substrates as described (Detarsio et al., 2003; Wheeler et al., 2005).

### Gel electrophoresis

Native PAGE was performed using a 5% (w/v) acrylamide separating gel. Electrophoresis was run for 90 min at 150 V at 4°C. NADP‐ME activity was determined by incubating the gels in a solution containing 50 mM Tris–HCl, pH 7.5, 10 mM L‐malate, 10 mM MgCl_2_, 0.5 mM NADP, 35 μg/mL nitroblue tetrazolium, and 0.85 μg/mL phenazine methosulfate at 30°C as described (Wheeler et al., 2005).

### Chlorophyll assay

To measure chlorophyll content, plants were grown in soil for 4 weeks in a growth chamber (HanKuk Scientific Instrument Company, HK‐GC800), under a 12/12 (light, 100 μmol m^−2^ s^−1^/dark) photoperiod at 23°C/21°C (day/night). For the chlorophyll assay, fresh leaves (50 mg) were ground in liquid nitrogen and extracted in 1 mL of 80% (v/v) acetone in the dark for 30 min. The supernatant was transferred to a new tube and the contents of chlorophyll A and B were measured using a Synergy HTX multimode microplate reader (BioTek). The chlorophyll concentrations were calculated as described (Ni et al., 2009).

### Leaf stomatal traits measurements

To measure stomatal size, the fourth fully expanded leaves were detached and immediately photographed to determine the total leaf area. The average number of stomata per unit area was obtained from three biological microscope (KOREA LAB TECH, KB‐300) images of each leaf, and the stomatal density per leaf was calculated. The stomatal length, width, and pore area were measured using ImageJ software on images acquired through biological microscopy.

### Gas‐exchange analysis

Gas‐exchange analysis was performed on plants grown in soil under 12 h/12 h (light, 100 μmol m^−2^ s^−1^/dark) cycles at 23°C/21°C (day/night). For measurements under non‐stressed conditions, plants were grown for 4 weeks and fully expanded fifth leaves were used. For well‐watered control conditions, two‐week‐old seedlings were maintained for an additional 4 weeks at 100% soil water‐holding capacity (SWC, well‐watered) or 50% SWC (chronic water‐deficit stress). Gas‐exchange measurements were therefore conducted on fully expanded fifth leaves of 6‐week‐old plants. Instantaneous WUE, net CO_2_ assimilation, stomatal conductance, and transpiration were measured using a LI‐6400XT gas analyzer (LI‐COR Biosciences, Lincoln, NE, USA) outfitted with the 6‐cm^2^ LI‐6400 chamber at CO_2_ value of 400 mmol. The measurement conditions were set at a reference photosynthetically active radiation (PAR) reference level of 1500 μmol m^−2^ s^−1^ and a leaf temperature of 23°C. The airflow rate was adjusted to 500 μmol s^−1^ to obtain constant relative humidity (~50%) and VPD values of ~1 kPa, which remained constant during the experiment.

### Water‐deficit stress assays

Plants were grown in square plastic pots (SIMPOL, Square Slit Planter 1) containing soil (Hanwol Bio) under a 12 h light/12 h dark photoperiod (100 μmol m^−2^ s^−1^) at 23°C/21°C (day/night). SWC was determined using a standard pot‐capacity method. Soil‐only control pots were filled with 140 g of dry soil, weighed, saturated with water, covered, and allowed to drain until cessation of dripping. Pots were then weighed again and the average weight (280 g per pot) was defined as 100% SWC. A pot weight of 210 g was defined as 50% SWC (Ginzburg et al., 2022; Lim et al., 2020). For the acute water‐deficit stress treatment, two‐week‐old seedlings were maintained under well‐watered conditions at 100% SWC. Acute water‐deficit stress was imposed by completely withholding irrigation for 14 days. Following the water‐deficit stress period, plants were rewatered and allowed to recover for 7 days and the survival rate was calculated.

For the chronic water‐deficit stress treatment, seedlings were grown for 2 weeks under well‐watered conditions and subsequently maintained for 8 weeks at either 100% SWC or 50% SWC until completion of the life cycle and seed set. During this period, soil‐only control pots were weighed every 3 days and water was added to restore the designated target weights. The percentage of bolting, soil water content, and the relative water content of leaves were monitored every week for 8 weeks. Stomatal parameters, gas‐exchange analyses, and survival rates were measured after 4 weeks of water treatments.

### H_2_O_2_ and lipid peroxidation activity

Hydrogen peroxide (H_2_O_2_) levels were quantified using the xylenol orange assay (Gay et al., 1999). Briefly, 100 μL of the supernatant was added to 1 mL of the assay solution containing 25 mM ferrous ammonium sulfate, 100 mM sorbitol, 125 μM xylenol orange, and 2.5 M sulfuric acid (H_2_SO_4_). The reaction mixture was incubated at room temperature for 45 min. Absorbance was then measured at 560 nm using a Synergy HTX multimode microplate reader (BioTek). The leaf samples were homogenized with 5 mL of 0.1% (w/v) trichloroacetic acid (TCA). The malondialdehyde (MDA) method was described by (Heath & Packer, 1968). After homogenization, the mixture was centrifuged at 12000 ***g*** for 10 min, and the supernatant was used for determining MDA content. To 1.0 mL of the supernatant, 4.0 mL of thiobarbituric acid (TBA) reagent was added, and the mixture was heated in a water bath at 95°C for 30 min. The reaction was then immediately cooled on ice. The cooled sample was centrifuged again at 10000 ***g*** for 10 min. The absorbance of the clear supernatant was recorded at 532 nm for MDA and the non‐specific absorbance at 600 nm. The non‐specific absorbance was subtracted from the 532 nm reading to obtain the specific MDA content.

### Total flavonoid measurement

The total flavonoid content (TFC) was determined using a modified version of the method originally described by (Zhishen et al., 1999). Briefly, 100 μL of *A. thaliana* leaf extract was mixed with 100 μL of 5% (w/v) sodium nitrite solution and 50 μL of distilled water. The mixture was incubated at room temperature for 6 min, after which 150 μL of 10% (w/v) aluminum chloride solution was added, followed by an additional 5‐min incubation. Next, 200 μL of 1 M sodium hydroxide solution was added and the reaction mixture was incubated for 1 h at 37°C. Absorbance was recorded at 510 nm using a multimode microplate reader (BioTek). A standard curve was constructed using a quercetin standard (Sigma‐Aldrich).

### RT‐qPCR of flavonoid genes

Seeds of the *McNADP‐ME1* overexpressing and EV control lines were germinated and grown in a growth chamber (HanKuk Scientific Instrument Company, HK‐GC800) modified under 12 h/12 h (light, 100 μmol m^−2^ s^−1^/dark) cycles at 23°C/21°C (day/night). Four‐week‐old plants were subjected to well‐watered and water‐deficit stressed conditions for 2 weeks. Total RNA was isolated using the Apure™ Plant SFGR RNA Kit (APBIO, CAT. H4005). Purified RNA samples were processed into single‐stranded cDNA using oligo (dT) primers according to the RevertAid First Strand cDNA Synthesis Kit (Thermo Fisher Scientific, K1622). The primers used for the qPCR experiment were designed using NCBI Primer‐BLAST and are listed in Table S1. Real‐time qPCR was performed using the Power SYBR Green PCR Master Mix (Thermo Fisher Scientific, CAT. 4367659). The following standard thermal profile was used for PCR reactions: 95°C for 10 min, 50 cycles of 95°C for 10 s, 60°C for 15 s and 72°C 15 s. Expression levels for flavonoid genes were normalized using the *A. thaliana TIP41‐like* (AT4G34270) gene as the standard.

### High‐light stress assays

*A. thaliana* plants were cultivated for 4 weeks under high‐light conditions with photosynthetically active radiation (PAR) of 350 μmol m^−2^ s^−1^ in a growth chamber (HanKuk Scientific Instrument Company, HK‐GC800). The photoperiod was set to 18/6 (light/dark) with day/night temperatures of 23°C/21°C.

### Statistical analysis

Statistical analysis was performed by GraphPad Prism software. All data were subjected to analysis of variance (ANOVA) followed by Dunnett's multi‐comparison test to compare all columns *vs*. WT in each condition. Values were expressed as the mean ± SE, and *P* value < 0.05 was considered as statistically significant.

## Author Contributions

SHK, JCC, and SDL conceived of the study, designed the research, and co‐wrote the paper. SHK, DYS, SYY, CHL, and SDL performed the experiments, data analyses, and data interpretation. All authors read and approved the final manuscript.

## Conflict of Interest

The authors declare no competing interests.

## Supporting information


**Table S1.** Table of Primer Sequences.

**Figure S1.** Characterization of the wild‐type, *CaMV35S::sGFP* empty vector control line and *McNADP‐ME1* overexpressing Arabidopsis lines. (a) Analysis of the *McNADP‐ME1* gene in Col‐0 wild‐type (WT), *CaMV35S::sGFP* empty vector control line and eight *McNADP‐ME1* overexpression lines (#1 to #8) by semiquantitative PCR. (b) Quantitative real‐time PCR analysis of *McNADP‐ME1* transcript abundance in wild‐type A. *thaliana* ecotype Col‐0,*CaMV35S::sGFP* empty vector line, and three independent *McNADP‐ME1* overexpressing lines (#2A, #3B, and #7A). Transcript levels of *McNADP‐ME1* in three different lines were quantified using *TIP41‐like* (AT4G34270) expression as a normalization standard. Values represent means ± SD of three biological replicates.

**Figure S2.** Subcellular localization of *McNADP‐ME1* genes expressed in *A. thaliana*. To identify subcellular localizations of ice plant *McNADP‐ME1* gene in A. *thaliana*. *Agrobacterium* harboring 35S::sGFP (EV control) and *35S::McNADP‐ME1‐sGFP* was transformed into *A. thaliana* and subcellular localization was determined by confocal microscopy. Bar = 20 μm.

**Figure S3.** *McNADP‐ME1* overexpression improves water‐deficit stress tolerance. (a) Representative images of three *McNADP‐ME1* overexpressing lines (#2, #3, and #7), wild‐type (col‐0, WT), and *CaMV35S::sGFP* empty vector (EV) control line. The two‐week‐old well‐watered plants were subjected to water‐deficit stress for 2 weeks and re‐watered for 7 days. (b) Survival rate (*n* = 3 replicates with average survival rate of 30 plants per replicate). (c) Fresh weight (*n* = 30 with three biological replicates). (d) Dry weight (*n* = 30 with three biological replicates). Values represent means ± SD, ns = non‐significant, and ***P < 0.001, one‐way ANOVA with Dunnett's multiple comparison test.

**Figure S4.** Assessment of stomata parameters in *McNADP‐ME1* overexpressing *Arabidopsis* under chronic water‐deficit conditions. Stomatal characteristics were analyzed in wild‐type (col‐0), empty vector (*CaMV35S::sGFP*), and McNADP‐ME1 overexpression lines (#2, #3, and #7) grown under well‐watered (100% soil water‐holding capacity) or chronic water‐deficit (50% SWC) conditions. Two‐week‐old well‐watered plants were grown for 4 weeks under water‐controlled conditions with different soil water‐holding capacity (100% or 50%) and stomatal length, width, and pore area were measured. (a) Representative images of stomatal aperture and density of the *CaMV35S::sGFP* (EV) control line and *McNADP‐ME1* overexpression line (#2). Red arrowheads indicate the position of stomata in the lower epidermis. (b) Stomatal length (*n* = 30 with three biological replicates). (f) Stomatal width (*n* = 30 with three biological replicates). (g) Stomatal pore area (*n* = 30 with three biological replicates). Values represent means ± SD, ns = non‐significant, **P < 0.01, and ***P < 0.001 one‐way ANOVA with Dunnett's multiple comparison test. Bar = 5 μm.

**Figure S5.** Gas‐exchange analysis of McNADP‐ME1 overexpressing *Arabidopsis* under chronic water‐deficit stress conditions. Stomatal characteristics were analyzed in wild‐type (col‐0), empty vector (*CaMV35S::sGFP*), and McNADP‐ME1 overexpression lines (#2, #3, and #7) grown under well‐watered (100% soil water‐holding capacity) or chronic drought (50% SWC) conditions. Two‐week‐old well‐watered plants were grown for 4 weeks under water‐controlled conditions with different soil water‐holding capacity and stomatal length, width, and pore area were measured. (a) Net CO_2_ assimilation (*n* = 10). (b) Stomatal conductance (*n* = 10). (c) Transpiration (*n* = 10). (d) instantaneous WUE (iWUE) (*n* = 10). Values represent means ± SD, ns = non‐significant, **P < 0.01, and ***P < 0.001 one‐way ANOVA with Dunnett's multiple comparison test.

**Figure S6.** High‐light stress phenotype of wild‐type, *CaMV35S::sGFP* empty vector control line and *McNADP‐ME1* overexpressing Arabidopsis lines. (a) Representative images of 28‐day‐old wild‐type (col‐0, WT), *CaMV35S::sGFP* empty vector (EV) control and three *McNADP‐ME1* overexpression lines (#2, #3 and #7). Scale bar, 5 cm. (b) Leaf number (*n* = 15 with three biological replicates). (c) Rosette diameter (*n* = 15 with three biological replicates). (d) Leaf fresh weight (*n* = 15 with three biological replicates). (e) Leaf dry weight (*n* = 15 with three biological replicates). Values represent means ± SD, ns = non‐significant, ***P < 0.001 one‐way ANOVA with Dunnett's multiple comparison test.

## Data Availability

The data that support the findings of this study are available on request from the corresponding author and in the supplementary information. The data are not publicly available due to privacy or ethical restrictions.
