# Supplementary material for: Overexpression of cytosolic NADP‐malic enzyme 1 from the common ice plant enhances water‐deficit and high‐light stress tolerance by modulating water‐use efficiency and flavonoid biosynthesis
Source: Plant J. 2026 Jun 6;126(5):e70968. doi: 10.1111/tpj.70968 (PMC13242266; doi:10.1111/tpj.70968)
Supplement: Supplementary file 2 — Figure S1. Characterization of the wild‐type, CaMV35S::sGFP empty vector control line and McNADP‐ME1 overexpressing Arabidopsis lines. (a) Analysis of the McNADP‐ME1 gene in Col‐0 wild‐type (WT), CaMV35S::sGFP empty vector control line and eight McNADP‐ME1 overexpression lines (#1 to #8) by semiquantitative PCR. (b) Quantitative real‐time PCR analysis of McNADP‐ME1 transcript abundance in wild‐type A. thaliana ecotype Col‐0,CaMV35S::sGFP empty vector line, and three independent McNADP‐ME1 overexpressing lines (#2A, #3B, and #7A). Transcript levels of McNADP‐ME1 in three different lines were quantified using TIP41‐like (AT4G34270) expression as a normalization standard. Values represent means ± SD of three biological replicates. [file TPJ-126-0-s007.docx]

**Supplementary Figure 1S.**

(a)

Relative expression

of *McNADP-ME1*

WT

EV

#2A

#3B

#7A

*OE-McNADP-ME1*

(b)


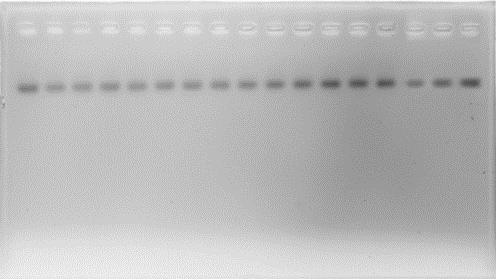

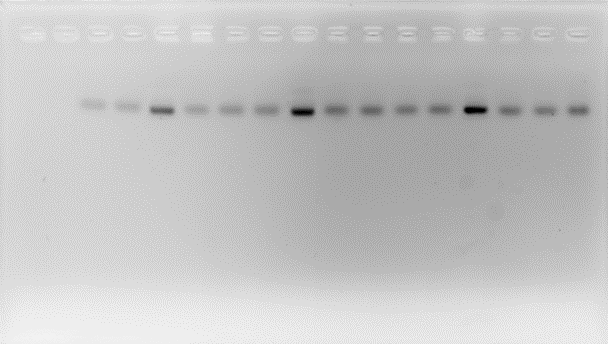


*McNADP-ME1*

*OE-McNADP-ME1*

*Tip41-like*

WT

EV

#2A

#3A

#7A

#1A

#1B

#2B

#2C

#3B

#4A

#5A

#6A

#6B

#7B

#7C

#8A

**Supplementary Figure S1. Characterization of the wild-type, *CaMV35S::sGFP* empty vector control line and *McNADP-ME1* overexpressing Arabidopsis lines.** (a) Analysis of the *McNADP-ME1* gene in Col-0 wild-type (WT), *CaMV35S::sGFP* empty vector control line and eight *McNADP-ME1* overexpression lines (#1 to #8) by semiquantitative PCR. (b) Quantitative real-time PCR analysis of *McNADP-ME1* transcript abundance in wild-type A. *thaliana* ecotype Col-0,*CaMV35S::sGFP* empty vector line, and three independent *McNADP-ME1* overexpressing lines (#2A, #3B, and #7A). Transcript levels of *McNADP-ME1* in three different lines were quantified using *TIP41-like* (AT4G34270) expression as a normalization standard. Values represent means ±SD of three biological replicates.
