# Supplementary material for: Overexpression of cytosolic NADP‐malic enzyme 1 from the common ice plant enhances water‐deficit and high‐light stress tolerance by modulating water‐use efficiency and flavonoid biosynthesis
Source: Plant J. 2026 Jun 6;126(5):e70968. doi: 10.1111/tpj.70968 (PMC13242266; doi:10.1111/tpj.70968)
Supplement: Supplementary file 3 — Figure S2. Subcellular localization of McNADP‐ME1 genes expressed in A. thaliana. To identify subcellular localizations of ice plant McNADP‐ME1 gene in A. thaliana. Agrobacterium harboring 35S::sGFP (EV control) and 35S::McNADP‐ME1‐sGFP was transformed into A. thaliana and subcellular localization was determined by confocal microscopy. Bar = 20 μm. [file TPJ-126-0-s004.docx]

**Supplementary Figure S2.**


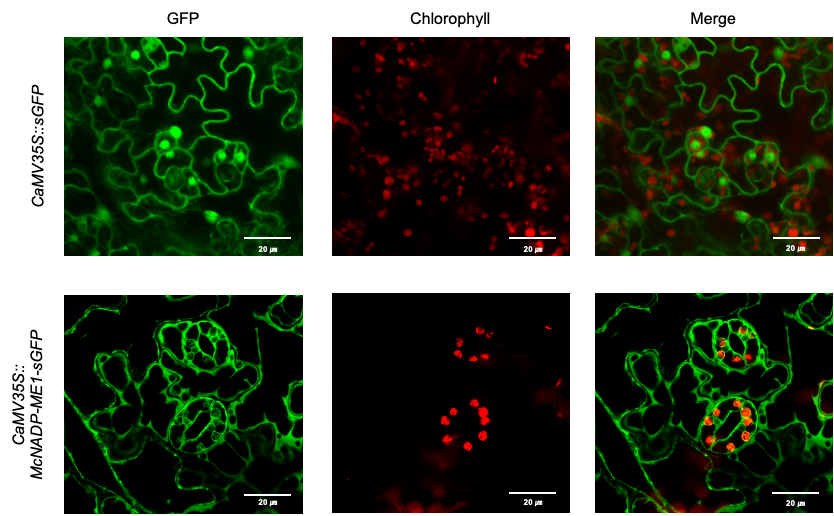


**Supplementary Figure S2. Subcellular localization of *McNADP-ME1* genes expressed in *A. thaliana*.** To identify subcellular localizations of ice plant *McNADP-ME1* gene in A. *thaliana.* *Agrobacterium* harboring 35S::sGFP (EV control) and *35S::McNADP-ME1-sGFP* was transformed into *A. thaliana* and subcellular localization was determined by confocal microscopy. Bar = 20 μm.
