# Supplementary material for: Overexpression of cytosolic NADP‐malic enzyme 1 from the common ice plant enhances water‐deficit and high‐light stress tolerance by modulating water‐use efficiency and flavonoid biosynthesis
Source: Plant J. 2026 Jun 6;126(5):e70968. doi: 10.1111/tpj.70968 (PMC13242266; doi:10.1111/tpj.70968)
Supplement: Supplementary file 4 — Figure S3. McNADP‐ME1 overexpression improves water‐deficit stress tolerance. (a) Representative images of three McNADP‐ME1 overexpressing lines (#2, #3, and #7), wild‐type (col‐0, WT), and CaMV35S::sGFP empty vector (EV) control line. The two‐week‐old well‐watered plants were subjected to water‐deficit stress for 2 weeks and re‐watered for 7 days. (b) Survival rate (n = 3 replicates with average survival rate of 30 plants per replicate). (c) Fresh weight (n = 30 with three biological replicates). (d) Dry weight (n = 30 with three biological replicates). Values represent means ± SD, ns = non‐significant, and ***P < 0.001, one‐way ANOVA with Dunnett's multiple comparison test. [file TPJ-126-0-s005.docx]

**Supplementary Figure S3.**


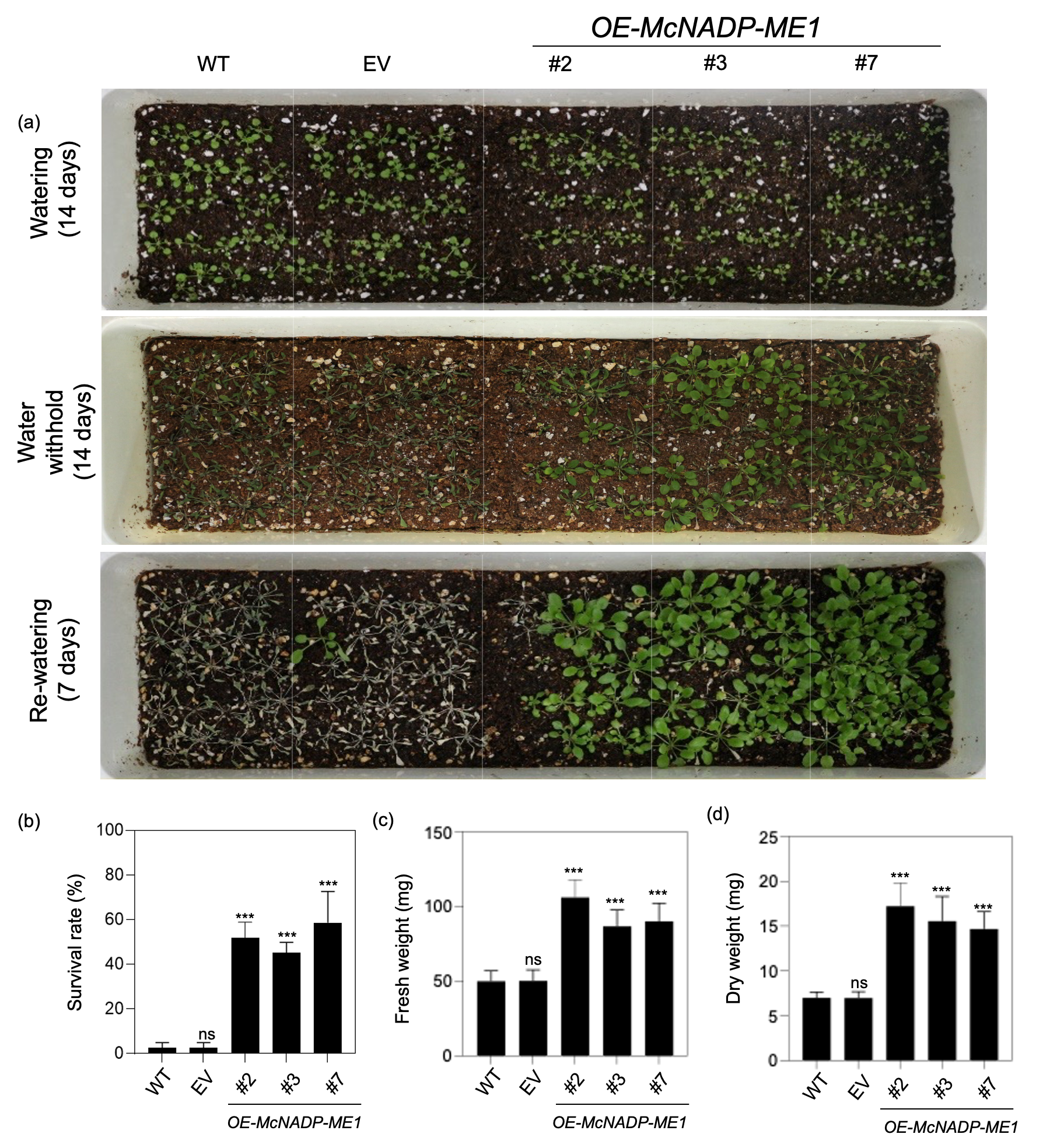


**Supplementary Figure S3. *McNADP-ME1* overexpression improves water-deficit stress tolerance**. (a) Representative images of three *McNADP-ME1* overexpressing lines (#2, #3, and #7), wild-type (col-0, WT), and *CaMV35S::sGFP* empty vector (EV) control line. The two-week-old well-watered plants were subjected to water-deficit stress for two weeks and re-watered for 7 days. (b) Survival rate (*n* = 3 replicates with average survival rate of 30 plants per replicate). (c) Fresh weight (*n* = 30 with three biological replicates). (d) Dry weight (*n* = 30 with three biological replicates). Values represent means ± SD, ns = non-significant, and ****p* < 0.001, one-way ANOVA with Dunnett's multiple comparison test.
