# Supplementary material for: Overexpression of cytosolic NADP‐malic enzyme 1 from the common ice plant enhances water‐deficit and high‐light stress tolerance by modulating water‐use efficiency and flavonoid biosynthesis
Source: Plant J. 2026 Jun 6;126(5):e70968. doi: 10.1111/tpj.70968 (PMC13242266; doi:10.1111/tpj.70968)
Supplement: Supplementary file 6 — Figure S5. Gas‐exchange analysis of McNADP‐ME1 overexpressing Arabidopsis under chronic water‐deficit stress conditions. Stomatal characteristics were analyzed in wild‐type (col‐0), empty vector (CaMV35S::sGFP), and McNADP‐ME1 overexpression lines (#2, #3, and #7) grown under well‐watered (100% soil water‐holding capacity) or chronic drought (50% SWC) conditions. Two‐week‐old well‐watered plants were grown for 4 weeks under water‐controlled conditions with different soil water‐holding capacity and stomatal length, width, and pore area were measured. (a) Net CO2 assimilation (n = 10). (b) Stomatal conductance (n = 10). (c) Transpiration (n = 10). (d) instantaneous WUE (iWUE) (n = 10). Values represent means ± SD, ns = non‐significant, **P < 0.01, and ***P < 0.001 one‐way ANOVA with Dunnett's multiple comparison test. [file TPJ-126-0-s006.docx]

**Supplementary Figure S5.**


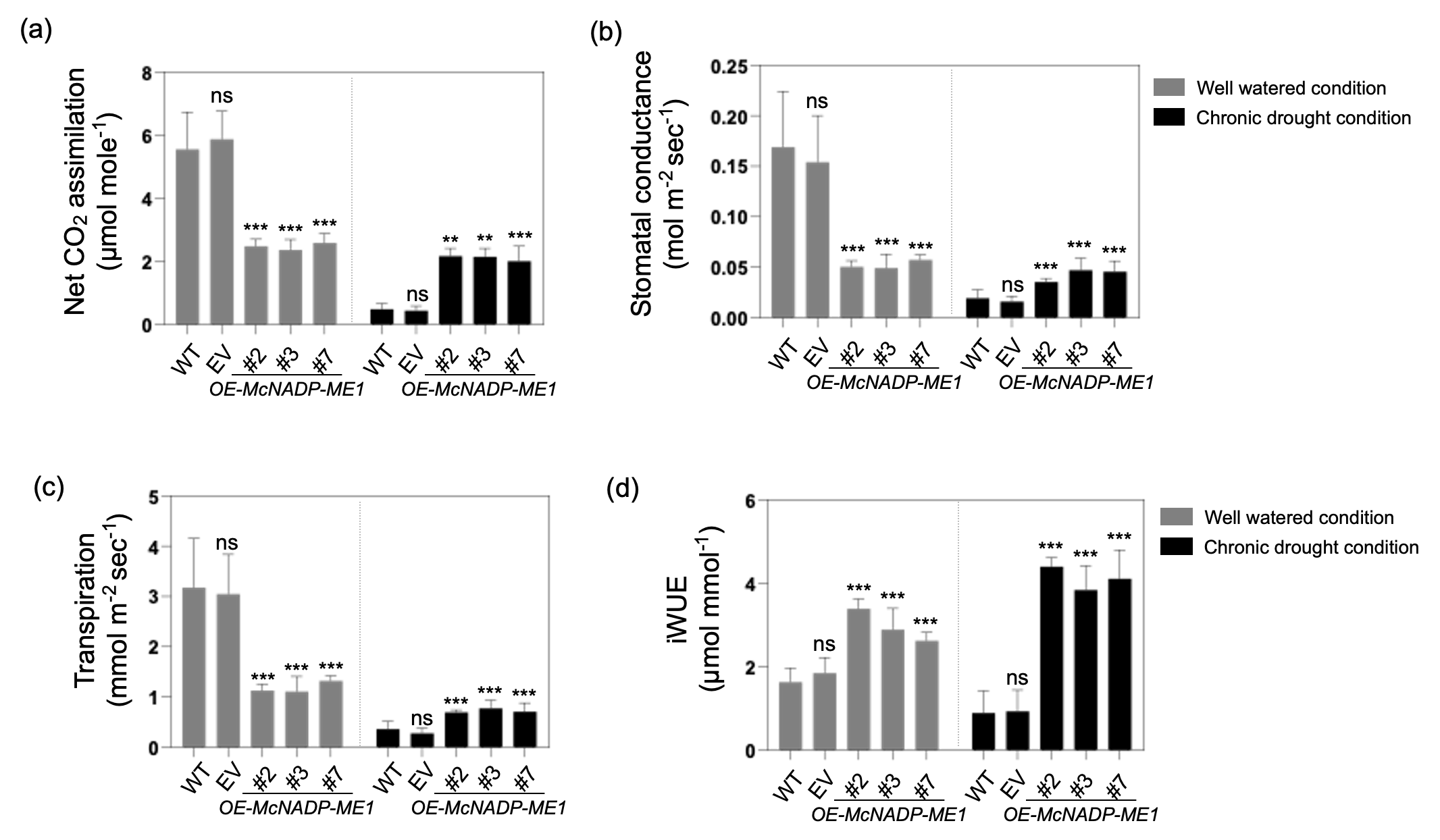


**Supplementary Figure S5. Gas-exchange analysis of McNADP-ME1 overexpressing *Arabidopsis* under chronic water-deficit stress conditions.** Stomatal characteristics were analyzed in wild-type (col-0), empty vector(*CaMV35S::sGFP)*, and McNADP-ME1 overexpression line (#2, #3, and #7) grown under well-watered (100% soil water-holding capacity) or chronic drought (50% SWC) conditions. Two-week-old well-watered plants were grown for 4 weeks under water-controlled conditions with different soil water-holding capacity and stomatal length, width, and pore area were measured. (a) Net CO_2_ assimilation (*n* = 10). (b) Stomatal conductance (*n* = 10). (c) Transpiration (*n* = 10). (d) instantaneous WUE (iWUE) (*n* = 10). Values represent means ± SD, ns = non-significant, ***p* < 0.01, and ****p* < 0.001 one-way ANOVA with Dunnett's multiple comparison test.
