# Supplementary material for: Overexpression of cytosolic NADP‐malic enzyme 1 from the common ice plant enhances water‐deficit and high‐light stress tolerance by modulating water‐use efficiency and flavonoid biosynthesis
Source: Plant J. 2026 Jun 6;126(5):e70968. doi: 10.1111/tpj.70968 (PMC13242266; doi:10.1111/tpj.70968)
Supplement: Supplementary file 7 — Figure S6. High‐light stress phenotype of wild‐type, CaMV35S::sGFP empty vector control line and McNADP‐ME1 overexpressing Arabidopsis lines. (a) Representative images of 28‐day‐old wild‐type (col‐0, WT), CaMV35S::sGFP empty vector (EV) control and three McNADP‐ME1 overexpression lines (#2, #3 and #7). Scale bar, 5 cm. (b) Leaf number (n = 15 with three biological replicates). (c) Rosette diameter (n = 15 with three biological replicates). (d) Leaf fresh weight (n = 15 with three biological replicates). (e) Leaf dry weight (n = 15 with three biological replicates). Values represent means ± SD, ns = non‐significant, ***P < 0.001 one‐way ANOVA with Dunnett's multiple comparison test. [file TPJ-126-0-s003.docx]

**Supplementary Figure S6.**


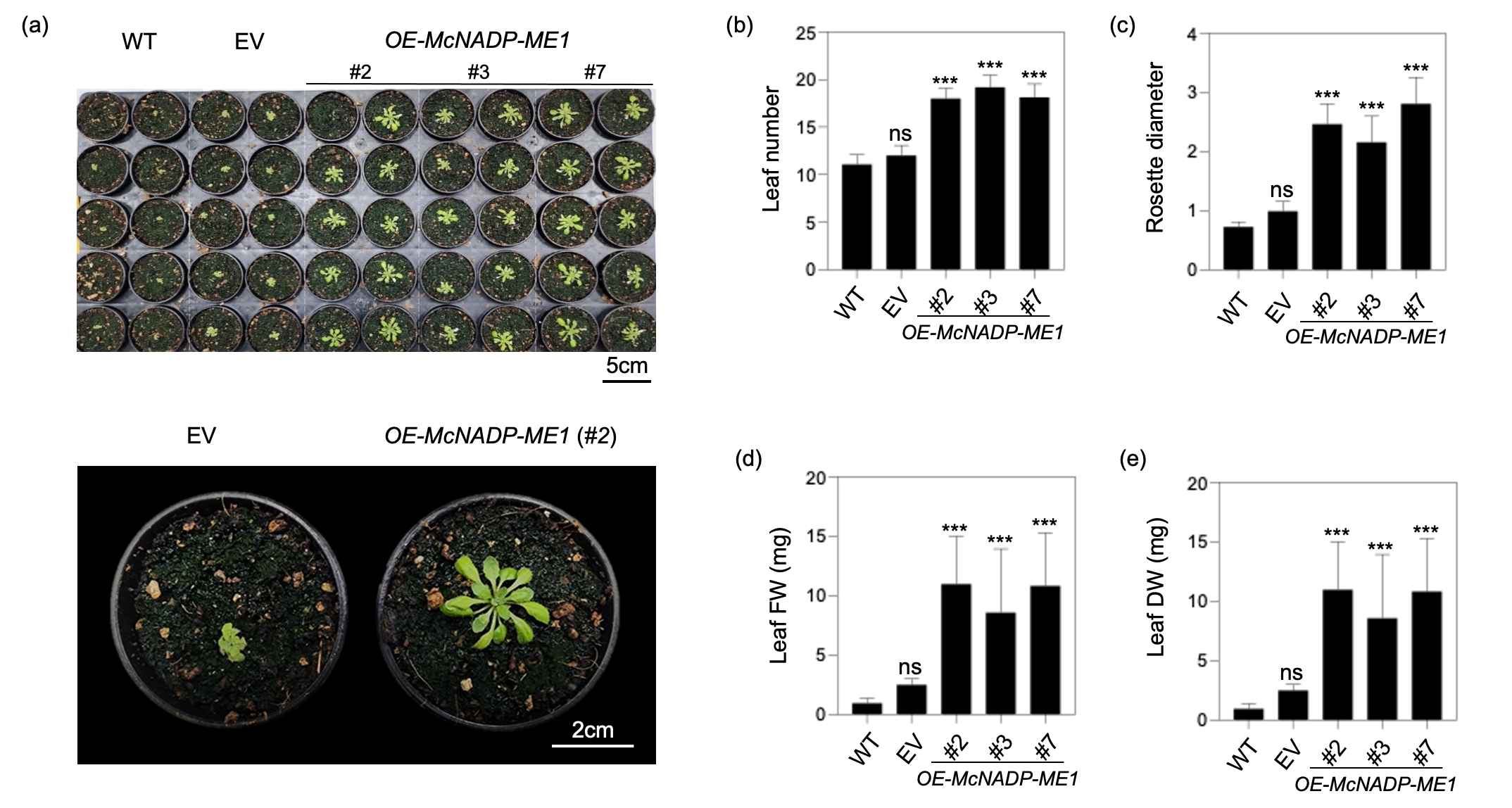


**Supplementary Figure S6. High-light stress phenotype of wild-type, *CaMV35S::sGFP* empty vector control line and *McNADP-ME1* overexpressing Arabidopsis lines.** (a) Representative images of 28-day-old wild-type (col-0, WT), *CaMV35S::sGFP* empty vector (EV) control and three *McNADP-ME1* overexpression lines (#2, #3 and #7). Scale bar, 5 cm. (b) Leaf number (*n* = 15 with three biological replicates). (c) Rosette diameter (*n* = 15 with three biological replicates). (d) Leaf fresh weight (*n* = 15 with three biological replicates). (e) Leaf dry weight (*n* = 15 with three biological replicates). Values represent means ± SD, ns = non-significant, ***P < 0.001 one-way ANOVA with Dunnett's multiple comparison test.
